# Rate of transcription elongation and sequence-specific pausing by RNA polymerase I directly influence rRNA processing

**DOI:** 10.1016/j.jbc.2022.102730

**Published:** 2022-11-22

**Authors:** Abigail K. Huffines, Krysta L. Engel, Sarah L. French, Yinfeng Zhang, Olga V. Viktorovskaya, David A. Schneider

**Affiliations:** 1 Department of Biochemistry and Molecular Genetics, University of Alabama at Birmingham, Birmingham, Alabama, USA; 2 Department of Microbiology, Immunology, and Cancer Biology, University of Virginia Health System, Charlottesville, Virginia, USA

**Keywords:** ribosome, transcription, transcription regulation, rRNA processing, RNA polymerase I, NET-Seq, ETS, external transcribed spacer, NET-Seq, native elongating transcript sequencing, Pol I, RNA polymerase I, rDNA, ribosomal DNA, TL, trigger loop

## Abstract

One of the first steps in ribosome biogenesis is transcription of the ribosomal DNA by RNA polymerase I (Pol I). Processing of the resultant rRNA begins cotranscriptionally, and perturbation of Pol I transcription elongation results in defective rRNA processing. Mechanistic insight regarding the link between transcription elongation and ribosome assembly is lacking because of limited *in vivo* methods to assay Pol I transcription. Here, we use native elongating transcript sequencing (NET-Seq) with a strain of *Saccharomyces cerevisiae* containing a point mutation in Pol I, *rpa190-F1205H*, which results in impaired rRNA processing and ribosome assembly. We previously demonstrated that this mutation caused a mild reduction in the transcription elongation rate of Pol I *in vitro*; however, transcription elongation by the mutant has not been characterized *in vivo*. Here, our findings demonstrate that the mutant Pol I has an increased pause propensity during processive transcription elongation both *in vitro* and *in vivo*. NET-Seq reveals that *rpa190-F1205H* Pol I displays alternative pause site preferences *in vivo*. Specifically, the mutant is sensitized to A/G residues in the RNA:DNA hybrid and at the last incorporated nucleotide position. Furthermore, both NET-Seq and EM analysis of Miller chromatin spreads reveal pileups of *rpa190-F1205H* Pol I throughout the ribosomal DNA, particularly at the 5′ end of the 35S gene. This combination of *in vitro* and *in vivo* analyses of a Pol I mutant provides novel insights into Pol I elongation properties and indicates how these properties are crucial for efficient cotranscriptional rRNA processing and ribosome assembly.

Ribosome biogenesis requires the intricate coordination of multiple biochemical processes. The first step is transcription by RNA polymerase I (Pol I), which synthesizes rRNA from a ribosomal DNA (rDNA) template. In *Saccharomyces cerevisiae* (yeast), Pol I transcribes a single rDNA gene, the 35S, which is organized in approximately 200 tandem repeats. Processing of the rRNA transcripts is complex and requires hundreds of transacting factors and RNAs, ultimately giving rise to the three largest mature rRNAs that serve as the backbone of the ribosome. The earliest studies on rRNA processing suggested that it could only occur post-transcriptionally, and this was supported by the detection of full-length rRNA products in the cell (1, 2). However, over the past 2 decades, it was shown that rRNA processing begins cotranscriptionally. The first evidence of cotranscriptional rRNA processing was from Miller chromatin spreads, which allow for the visualization of engaged Pol I transcription elongation complexes with a DNA template *in vivo*. Findings from that study demonstrated that nascent transcripts were cotranscriptionally cleaved while Pol I was still associated with the rDNA (3, 4). Furthermore, it was discovered that when transcription elongation by Pol I is perturbed, there is a corresponding disturbance in rRNA processing and ribosome biogenesis (5). Collectively, these findings demonstrate that cotranscriptional processing is important for the efficient maturation of rRNAs, but the regulation of this process is not well defined.

The mechanism by which transcription elongation kinetics influences the processing of nascent RNA is not entirely clear. One model proposes that the rate of RNA synthesis may impact the folding of RNA and therefore, the kinetics of cotranscriptional events that occur, such as processing (reviewed in Refs. (6, 7, 8)). Furthermore, a recent publication reported that there are sequence-specific effects on Pol I transcription rate and rRNA folding (9). Those results demonstrated that Pol I occupies G/C-rich rDNA at a higher frequency compared with A/T-rich rDNA, and that weak rRNA structures that form behind the polymerase may allow for more backtracking, which could slow transcription down. Those findings and others (10) suggest that polymerase pausing influences the kinetics of RNA folding and possibly vice versa. In addition, the efficiency of RNA folding has been shown to have an effect on Pol II transcription rate (11), suggesting that perhaps the coupling of elongation rate, RNA processing, and RNA folding is conserved across the three Pols or at least Pols I and II.

A number of studies have described the impact of altered polymerase elongation properties on RNA processing, and this has been directly observed for Pol I. Previously, it was established that the mutation of one of the aspartate residues that coordinate Mg^2+^ in the second largest subunit of Pol I, *rpa135-D784G*, impaired elongation rate *in vitro* and caused defects in rRNA processing and ribosome biogenesis (5). However, the complete characterization of Pol I transcription elongation and how the disruption of these properties can lead to defects in rRNA maturation and ribosome assembly is lacking *in vivo*. The cotranscriptional nature of rRNA processing, as well as the previous identification of elongation-defective Pol I mutants, makes this an exceptional system to investigate the dependence of cotranscriptional events on polymerase elongation properties ((3, 4, 12) and reviewed in Ref. (13)).

To explore the connection between Pol I elongation kinetics and cotranscriptional events further, we characterized the effects of a Pol I mutant that contains a point mutation within the trigger loop (TL) region. The TL is a conserved domain within the active site of multisubunit Pols across all domains of life and is known to directly affect several properties, including transcription elongation rate and pausing propensity (reviewed in Refs. (14, 15, 16)). We have previously shown that this mutation, *rpa190-F1205H*, reduced elongation rate by Pol I *in vitro* (17). Here, we thoroughly characterize the transcription elongation efficiency of the WT and mutant polymerases using single-turnover *in vitro* transcription assays with a selection of rDNA templates. These assays revealed sequence-specific pause sites for the WT enzyme and enhanced pausing by the mutant. To characterize pausing *in vivo*, we used two separate methods: native elongating transcript sequencing (NET-Seq) and EM of Miller chromatin spreads. These complementary methods reveal that this mutation induces significant changes to Pol I occupancy patterns throughout the 35S gene, which could be responsible for the observed dramatic changes in rRNA processing and subsequent impairments of ribosome biogenesis.

Collectively, these data suggest that increased pausing by Pol I results in aberrant cotranscriptional processing (possibly because of changes in rRNA secondary structure) and ribosome assembly events. These results support the model that transcription elongation is an important aspect of gene expression that not only influences the quantity but also the quality of the RNA that is produced. Since both the WT and *rpa190-F1205H* polymerases display sensitivities to particular sequences, our findings also demonstrate that Pol I has evolved a unique set of transcription elongation properties that respond to the rDNA sequence to promote efficient ribosome biogenesis.

## Results

### The *rpa190-F1205H* mutation induces sequence-specific pausing by Pol I *in vitro*

**Figure 1:**
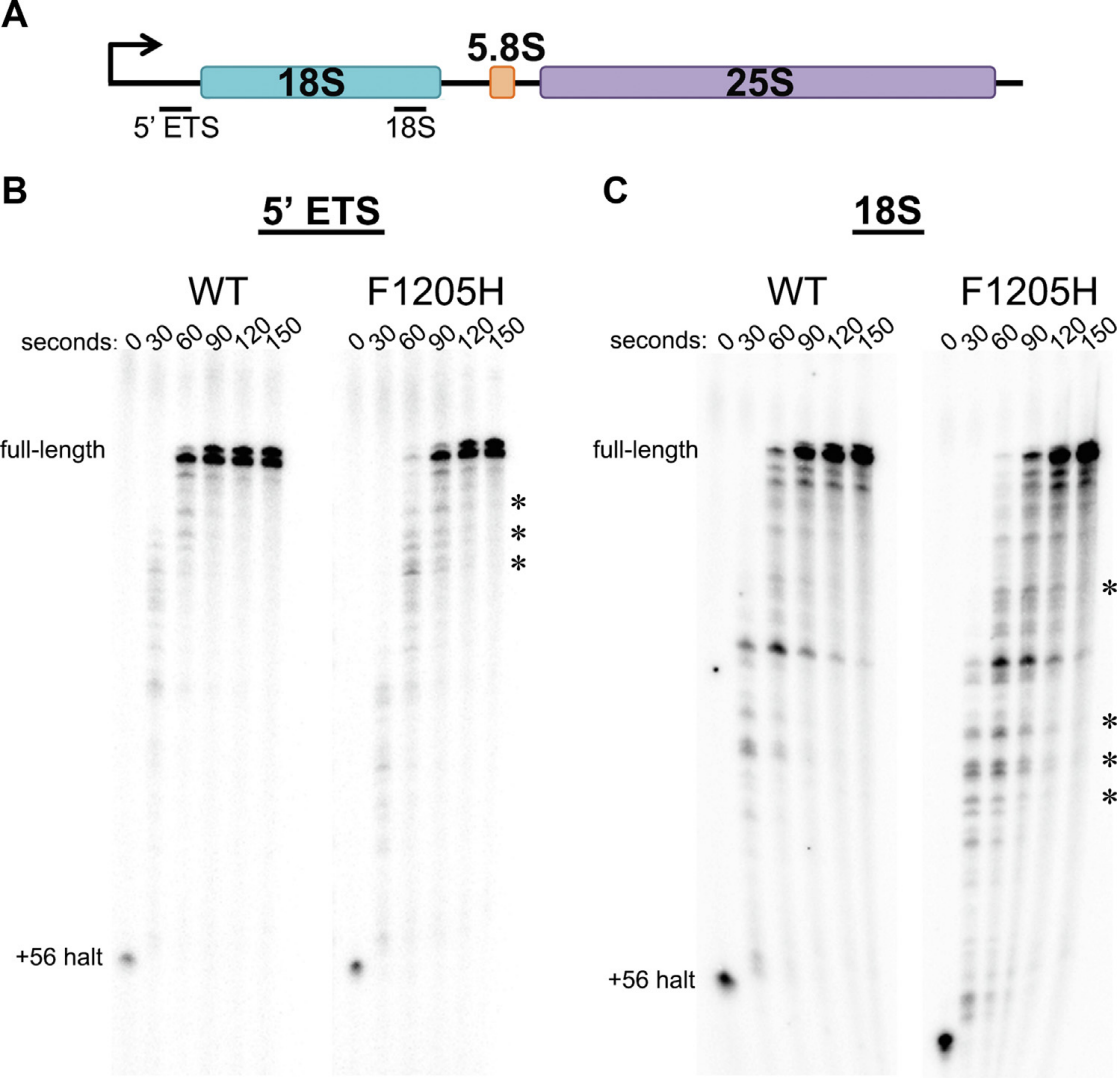
***rpa190-F1205H* Pol I has an increased pause propensity *in vitro*.** Purified *rpa190-F1205H* and WT Pol I were used to determine the pause propensity *in vitro*. The reactions were performed using 10 μM NTPs, and the RNA transcripts were labeled using [α-^32^P] GTP. *A*, a schematic (drawn to scale) indicating the positions of individual templates used for transcription in *B* and *C*. *B* and *C*, *asterisks* denote sites of polymerase pausing that are exaggerated by the *rpa190-F1205H* mutation. Assays on each template were performed at least four times, with representative gels shown for each. Pol I, polymerase I.

To investigate the effect of rDNA sequence elements on Pol I activity, we focused on a phenylalanine to histidine point mutation in a highly conserved position in the TL, *rpa190-F1205H*, and assayed the activity of this enzyme as compared with WT in single-turnover *in vitro* transcription assays. We have shown previously that the transcription elongation rate of *rpa190-F1205H* Pol I is ∼40% of the WT rate (17); however, this enzyme has not yet been fully characterized. For templates in these reactions, we used two different regions of the *S. cerevisiae* rDNA locus that were cloned downstream of the Pol I core promoter (see details in the Experimental procedures section and Ref. (5)). Downstream of the promoter in both templates, we included a 55-nucleotide region that does not encode any C residues in the nontemplate strand. Thus, we initiated transcription by incubation with 10 μM ATP, GTP, and UTP, resulting in the synchronization of elongation complexes at the first encoded C residue (at position +56, relative to the transcription start site; (17, 18)). To measure transcription elongation properties of the polymerases, 10 μM CTP was added to the reactions, and samples were collected as a function of time. We observed obvious sequence-specific pausing by WT Pol I at several regions within the rDNA templates (observed as distinct bands of lower molecular weight that appear and dissipate over the time course; Fig. 1). The simplest interpretation for the varied band intensities of these pauses is that the propensity for Pol I to enter the pause and escape it are based on the sequence, and some sequences confer a longer pause than others. These results demonstrate that Pol I is prone to sequence-specific pausing within the rDNA template. Since these reactions were performed with only the essential purified proteins required for *in vitro* transcription, these pause events are likely not dependent on trans-acting protein factors.

On each template, the *rpa190-F1205H* Pol I required slightly more time to produce full-length runoff product than WT Pol I, which is consistent with our previous studies demonstrating that the mutant enzyme has a reduced elongation rate (17). In addition to our previous studies showing that there was a reduction in the overall rate of transcription elongation by the mutant Pol I, here, we observed altered pausing by the *rpa190-F1205H* enzyme. Specifically, the duration of several pause sites was lengthened when transcribed by the mutant polymerase (indicated by *asterisks*, Fig. 1, *B* and *C*). These data demonstrate that the mutation of the TL in Pol I induces pausing by the enzyme on the rDNA sequence, relative to WT Pol I, even in a highly purified system.

### *rpa190-F1205H* affects Pol I activity and promotes pausing *in vivo*

The *in vitro* biochemical data demonstrate that the *rpa190-F1205H* mutation not only reduces Pol I transcription elongation rate (17) but also affects polymerase pausing on the native rDNA sequences. To determine whether this phenomenon is also observed *in vivo*, we performed EM of Miller chromatin spreads and NET-Seq. These techniques allow for the direct visualization of Pol I transcription on individual rDNA repeats (Miller chromatin spreads) as well as precise mapping of the positioning of individual Pol I molecules throughout the rDNA at single nucleotide resolution (NET-Seq).

**Figure 2:**
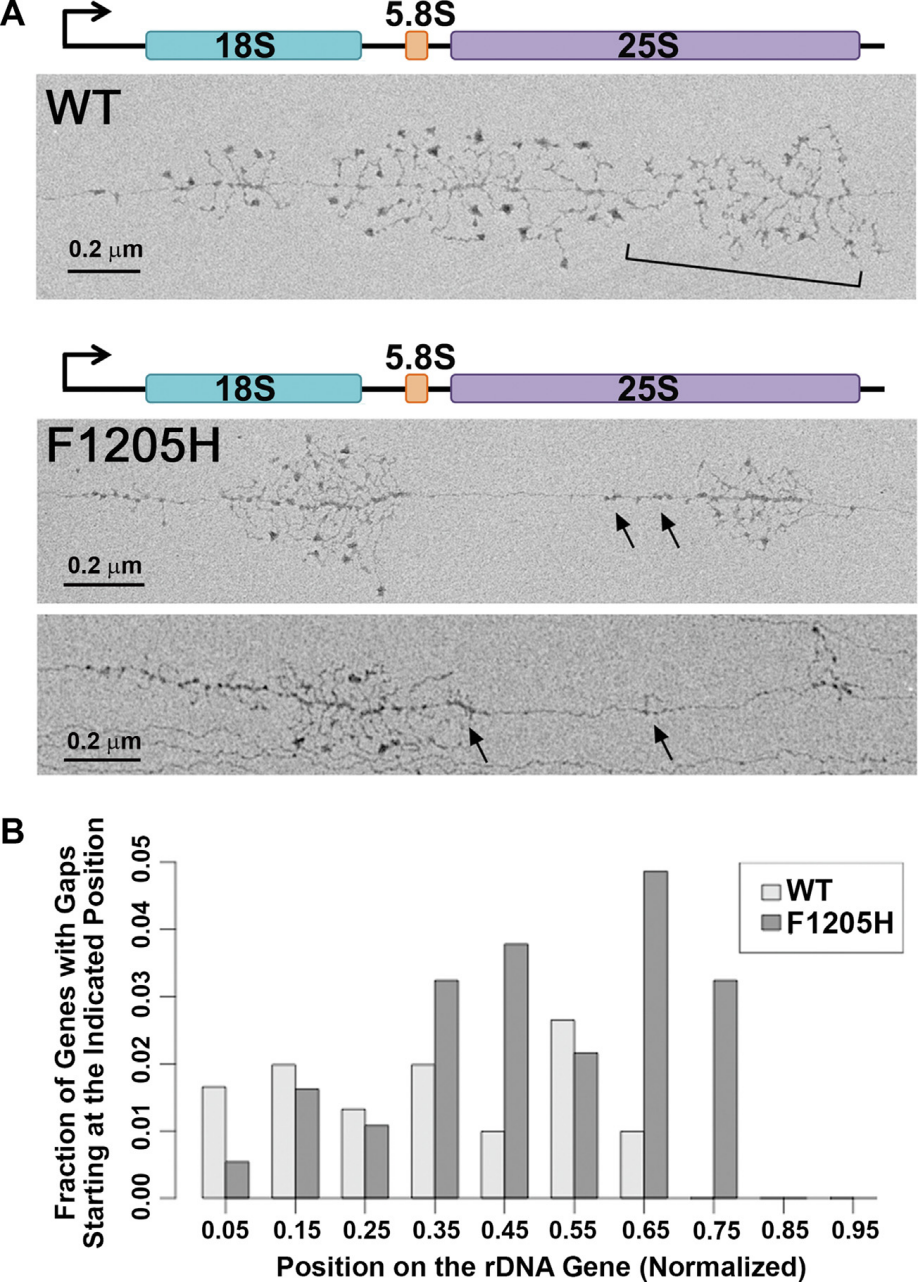
**Miller chromatin spreads identify defects in Pol I distribution on the rDNA in *rpa190-F1205* yeast.***A*, Miller chromatin spreads of WT and *rpa190-F120*5*H* yeast strains. The representative WT rDNA gene shows the typical features of Pol I transcription, including high polymerase density, relatively uniform distribution of transcripts across the gene, and nascent transcript cleavage (cleaved transcripts indicated by *bracket*). The representative *rpa190-F1205H* genes show large polymerase-free gaps, a higher polymerase density at the 5′ end of the genes, and some evidence for nascent transcript degradation (in regions denoted by *arrows*). See Fig. S1 for comparison of these three rDNA repeats with additional repeats from the *rpa190-F1205H* strain. *B*, positions of large polymerase-free gaps observed in chromatin spreads from WT and *rpa190-F1205H* yeast. The frequency of start sites (promoter-proximal ends) of gaps large enough to encompass at least 40 polymerases is plotted *versus* position along a normalized rDNA gene. The absence of start sites for gaps at the 3′ end of the gene results from the fact that only large gaps representing a quarter of the gene were scored. Data for the WT strain have been reported previously (19). Pol I, polymerase I; rDNA, ribosomal DNA.

Electron micrographs of Miller chromatin spreads reveal snapshots of rDNA transcription *in vivo*. We and others have previously used this technique to evaluate a number of features of Pol I transcription, including polymerase pausing and nascent transcript processing (4, 17, 19, 20). Historically, the analysis of Miller chromatin spreads has played a key role in demonstrating that rRNA processing and preribosome assembly begins cotranscriptionally (3, 4, 5). Therefore, this is a valuable tool to closely investigate features of transcription elongation by Pol I. Since Pol I is densely packed on the active rDNA repeats, the effects of pausing are readily observed. If one polymerase enters a paused state while the enzymes downstream continue to elongate, an extended gap between polymerases is evident. Therefore, the presence of such gaps suggests an increase in Pol I pausing during transcription. Long gaps have been observed at a relatively low frequency in WT cells (19). We examined Miller chromatin spreads of rDNA from WT and *rpa190-F1205H* yeast strains to evaluate the effects of the mutation on Pol I activity *in vivo* (Figs. 2*A* and S1). The analyzed Pol I distribution on the rDNA template from the *rpa190-F1205H* strain appeared strikingly different than those from WT yeast. First, large polymerase-free gaps were frequently observed on the 35S gene in the mutant, indicating an increased pause propensity for Pol I in the mutant *versus* WT strain. Second, many analyzed Miller spreads had fewer Pol I elongation complexes in the 3′ portion of the rDNA gene relative to the 5′ portion. Finally, we observed that nascent transcripts appeared shorter in the mutant as compared with WT, particularly at the 3′ end of the rDNA template. The simplest explanation for this qualitative observation is that the rRNA may be degraded by endonucleases or exonucleases cotranscriptionally.

To quantify potentially long pauses, we measured the frequency of large gaps between transcription elongation complexes (>23% of the 35S gene length; as performed previously (19)). Large gaps occurred at nearly twice the frequency in the mutant as compared with WT (21% of 185 35S genes analyzed and 12% (19) of 302 35S genes analyzed, respectively; Fig. 2*B*). We observed more gaps in the downstream half of the rDNA repeats in the mutant as compared with WT. In addition, there was a reduction in gaps at the 5′ end of the rDNA template in the *rpa190-F1205H* strain, suggesting that Pol I transcription elongation rate may be uniformly slower and pausing may be increased at the beginning of the 35S gene. Finally, the *rpa190-F1205H* Pol I appears to be more likely to accumulate in dense clusters on the rDNA template compared with WT Pol I, which is distributed more evenly across the entire 35S gene. Given that each active yeast rDNA repeat has a large number of engaged Pol I elongation complexes, these dense clusters could represent polymerase “traffic jams,” with *rpa190-F1205H* Pol I complexes becoming backed up on the template because of increased pausing. This *in vivo* evidence for pausing is consistent with *in vitro* observations (Fig. 1).

### NET-Seq reveals that *rpa190-F1205H* Pol I is repositioned on the rDNA

To further investigate the *in vivo* effects on transcription conferred by the *rpa190-F1205H* mutation, we used NET-Seq. This technique was first developed only about a decade ago to examine transcription by Pol II at high resolution *in vivo* (21). Our laboratory adapted these methods to investigate the properties of transcription by Pol I (22), and we have previously used it to demonstrate the role of various factors in transcription elongation (23, 24) and to characterize the mechanism of action of a small-molecule Pol I inhibitor *in vivo* (25). Therefore, this is a powerful tool to interrogate features of transcription by Pol I, especially when characterizing a mutation such as *rpa190-F1205H*. For these experiments, we generated triplicate NET-Seq libraries for both the WT and *rpa190-F1205H* yeast strains. We mapped the resultant reads back to the yeast genome and plotted the polymerase position on the rDNA for three replicates for each strain (Fig. S2, *left* and *middle panels* of *A* and *B*). Spearman correlation coefficient values were generated (Fig. S2, *right panels* of *A* and *B*) to determine the similarity between replicates (as described previously (22, 23, 24, 25)). Consistent with previous publications from our laboratory (22, 23, 24, 25), we found that within strains, Pol I occupancy was highly reproducible for both WT and *rpa190-F1205H* yeast (indicated by similar overlays in the histograms and Spearman correlation coefficient values >0.9). We performed principal component analysis (Fig. S3), which supported this conclusion, as the WT and mutant samples clustered away from each other, and the majority of the divergence (over 94%) between all samples was described by PC1, which represented the variation between WT and mutant libraries.

**Figure 3:**
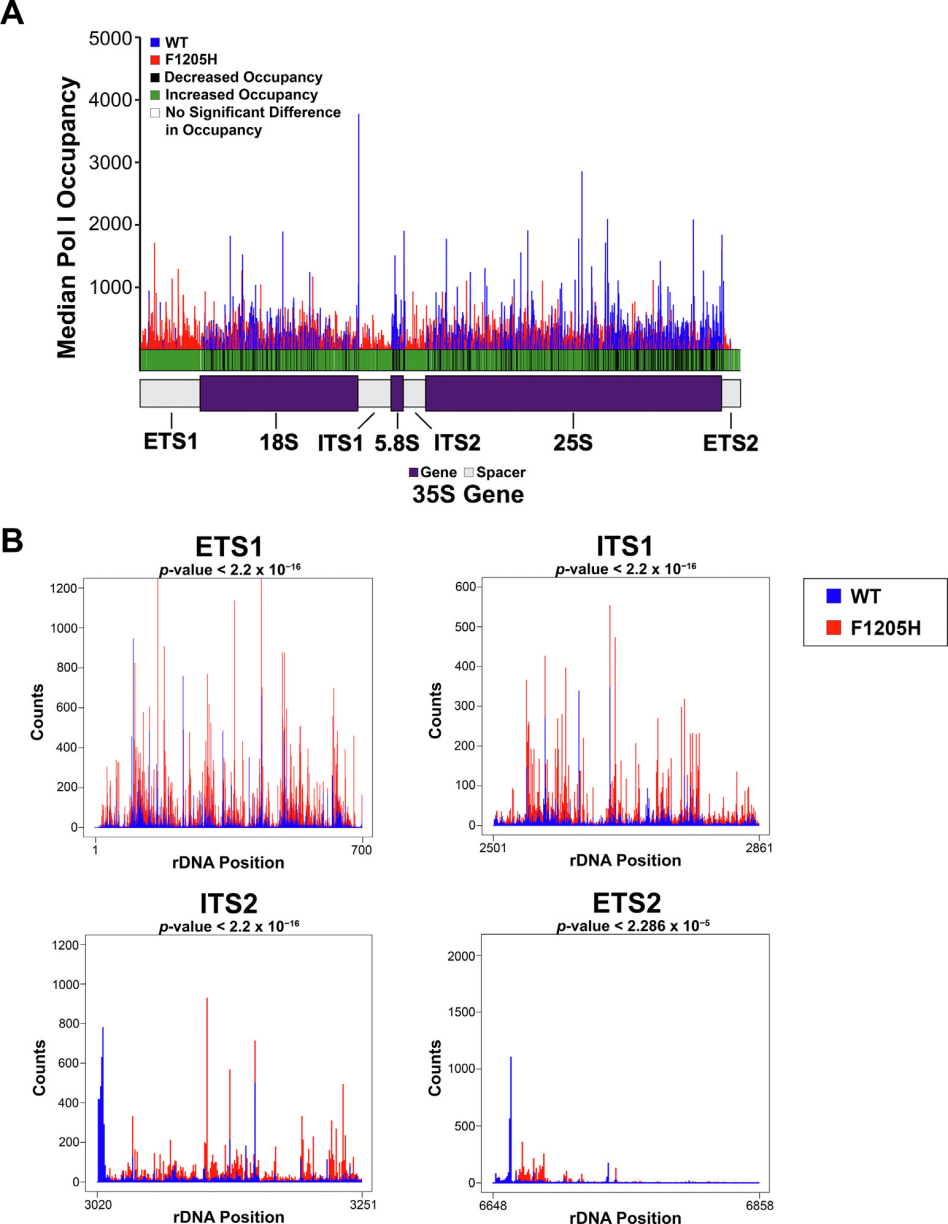
**NET-Seq indicates an altered pause profile in *rpa190-F1205H* yeast.***A*, the median Pol I occupancy in the WT (*blue*) and *rpa190-F1205H* (*red*) strains was mapped to the rDNA gene. At each position, a *t* test was executed to determine whether there was a significant difference in occupancy (*p* < 0.05) between strains. These results (*green* [increased], *black* [decreased], and *white* [no change]) are indicated below the histogram for the *rpa190-F1205H* strain with respect to WT. *B*, histograms depicting median Pol I occupancy differences in the spacer regions only (ETS1, ITS1, ITS2, and ETS2). The Kolmogorov–Smirnov (K–S) test was used to determine whether the patterns in each strain were from the same distribution (see *p* values located above each graph). ETS, external transcribed spacer; ITS, internal transcribed spacer; NET-Seq, native elongating transcript sequencing; Pol I, polymerase I; rDNA, ribosomal DNA.

After confirming that Pol I occupancy was reproducible within strains, we further examined occupancy patterns between strains. The median Pol I occupancy for each strain was plotted along the rDNA repeat, and a *t* test was performed for the median Pol I count at each individual rDNA position between strains to determine whether there was a significant difference in the *rpa190-F1205H* strain as compared with WT (Fig. 3*A*). Strikingly, there was a significant difference in occupancy across the 35S gene between strains, as indicated by the colors included below the histogram (*green* for increased occupancy and *black* for decreased occupancy in the mutant as compared with WT). From these NET-Seq data, we cannot draw definitive conclusions about the kinetics of Pol I transcription since each experiment represents a snapshot. However, we can interpret highly occupied regions as areas where Pol I is potentially paused on the template or is transcribing more slowly than positions showing lower occupancy, which could be interpreted as areas where Pol I is traversing the rDNA template more rapidly. In addition, very low regions of occupancy could indicate that the polymerases have prematurely disengaged with the template, especially if they are widespread. Figure 3*A* suggests that in the *rpa190-F1205H* strain, there is a significant increase in Pol I occupancy as compared with WT, and this increase seems more exaggerated at the 5′ end of the 35S gene (see the external transcribed spacer 1 [ETS1]) as compared with the 3′ end (see the external transcribed spacer 2 [ETS2]). This increase in occupancy is especially noticeable in the spacer regions (as evidenced by the almost *solid green markers* in those regions in Fig. 3*A* *versus* the mixture of *black* and *green markers* in the gene regions), though it is present across the length of the entire gene in every region. This is likely because of unavoidable mature rRNA product contamination in the NET-Seq samples, a limitation of this experiment that we have discussed in a previous publication (25). During rRNA synthesis, the spacer regions are rapidly cotranscriptionally and post-transcriptionally processed and removed prior to rRNA maturation (2, 4, 5, 12). Therefore, evaluation of Pol I occupancy in the spacer regions allows for the direct measure of nascent rRNA, without interference from contaminating mature rRNA. Therefore, we investigated the occupancy patterns in these four spacer regions (ETS1, internal transcribed spacer 1, internal transcribed spacer 2, and ETS2) more extensively to examine occupancy patterns and diminish mature product contamination as much as possible. Figure 3*B* indicates that there is an obvious increase in occupancy in the ETS1 and internal transcribed spacer 1 (the most 5′ regions) in the *rpa190-F1205H* strain as compared with WT, with a modest occupancy increase in the internal transcribed spacer 2 region and some occupancy differences present in the most 3′ region, the ETS2. In addition, the Kolmogorov–Smirnov test was used for statistical comparison in these regions, where a *p* value <0.05 indicates that the patterns between strains are not from the same distribution. The Kolmogorov–Smirnov test demonstrated that the distribution patterns were significantly different between strains in all four spacer regions. The occupancy differences observed in Figure 3 were validated by generating moving average and cumulative distribution function plots (Figs. S4 and S5, respectively). Collectively, these data suggest that consistent with Figure 2, *rpa190-F1205H* Pol I pauses more frequently than WT Pol I *in vivo*, especially at the 5′ end of the 35S gene, evidenced by increased occupancy.

**Figure 4:**
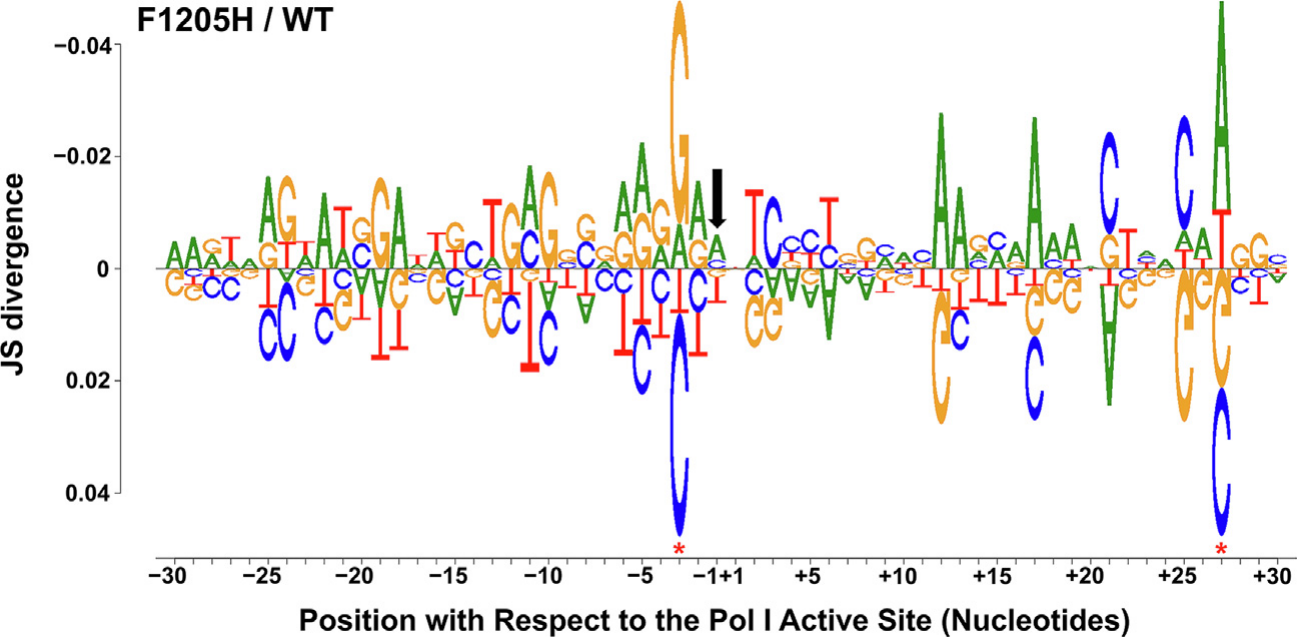
**The *rpa190-F1205H* mutation alters the nucleotide preference at pause sites *in vivo*.** A DiffLogo plot is shown, which demonstrates the sequence preference differences for the top 2.5% occupied positions in the spacer regions only for the *rpa190-F1205H* strain (*top*, JS divergence <0) and the WT strain (*bottom*, JS divergence >0). The *black arrow* indicates the position of the last incorporated nucleotide into the transcript. *Red asterisks* indicate positions of significance (*p* < 0.05), indicating that the sequences at that position are not from the same distribution. JS, Jensen–Shannon.

To gain insight into features of the rDNA that influence the strain-altered Pol I occupancy patterns shown in Figure 3, we took advantage of the high-resolution nature of NET-Seq data and determined the sequence preferences for each strain by generating a DiffLogo (Fig. 4). The top 2.5% occupied positions in the spacer regions were identified in both strains, and the highly occupied sequences were displayed for the mutant strain on top (Jensen–Shannon divergence <0), compared with the WT strain below (Jensen–Shannon divergence >0). Just like in Figure 3*B*, only the spacer regions were included in this analysis to reduce sequence enrichments coming from mature rRNA product contamination. Figure 4 demonstrates that there is polymerase repositioning on the rDNA template between strains, as *rpa190-F1205H* Pol I readily occupies A/G-rich regions that are present in the RNA:DNA hybrid, as compared with the C/T-rich regions occupied by WT Pol I. The RNA:DNA hybrid contributes directly to the stability of transcription elongation complexes (26, 27), so changes in sequence preferences in the hybrid region could directly impact pausing and nucleotide addition. Altogether, these NET-Seq results suggest that *rpa190-F1205H* Pol I exhibits impaired transcription elongation as compared with WT Pol I *in vivo*, and these findings could explain some of the pausing effects seen in Figures 2 and 3.

### rRNA processing and ribosome biogenesis are defective in *rpa190-F1205H* yeast

**Figure 5:**
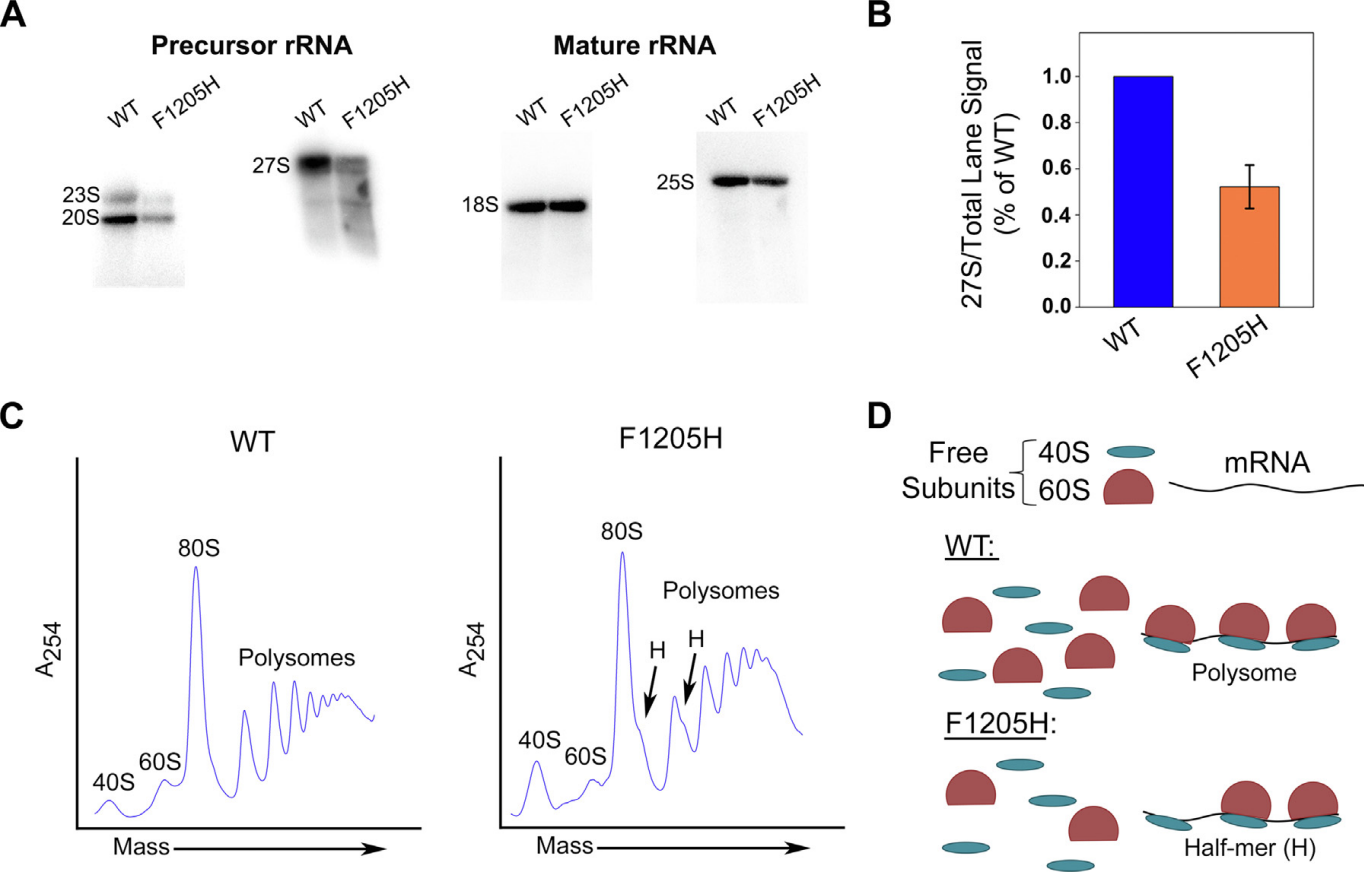
**The *rpa190-F1205H* mutation induces rRNA processing and ribosome biogenesis defects.***A*, Northern blot analysis of precursor and mature rRNAs. The *rpa190-F1205H* mutation leads to a loss of the precursor rRNAs. *B*, degradation of the 27S species is observed as the smear of signal below the full-length 27S. The degradation of 27S was quantified by measuring the ratio of full-length 27S divided by the total lane signal (n = 4). *C*, polysome profile analysis (sucrose gradient centrifugation) was performed on WT and mutant strains. Free ribosomal subunits (40S and 60S) were separated from monosomes (80S) and polysomes. Half-mers (H) were detected in the *rpa190-F1205H* strain, along with an altered relative abundance of 60S. The traces shown here are representative of three biological replicates (n = 3). *D*, schematic indicating how a half-mer is formed.

Given the previously characterized relationship between Pol I elongation defects and rRNA processing and ribosome biogenesis (5), we hypothesized that Pol I elongation rate and pausing frequency influence these cotranscriptional processes. Figure 1, Figure 2, Figure 3, Figure 4 demonstrate that *rpa190-F1205H* Pol I exhibits transcriptional defects and increased pausing both *in vitro* and *in vivo*. Therefore, we tested our hypothesis by determining whether ribosome biogenesis was impaired in the *rpa190-F1205H* yeast strain. We used Northern blot analysis to measure the level of precursor and mature rRNAs in both strains. In the *rpa190-F1205H* strain, we observed a reduction in the level of precursor rRNAs for both the 18S and 25S (Fig. 5*A*). This loss of signal was most likely because of degradation of the pre-rRNAs. However, since exosome-mediated RNA decay occurs in the 3′ to 5′ direction and we are confined to using a Northern probe that targets the 3′ end of the species (Fig. S6), degradation of the 23S and 20S pre-rRNAs is not detectable in this experiment. Alternatively, the 5′ location of the 27S probe yielded clear evidence for the degradation of this rRNA species in the mutant strain but not in WT (Figs. 5*A* and S6). Quantification of this degradation was performed by measuring the ratio of the amount of full-length 27S relative to total signal within the lane. These results identified significant degradation of the 27S precursor in the mutant *versus* WT (Fig. 5*B*). To demonstrate that these processing defects are a direct result of perturbations in transcription elongation, we previously analyzed processing intermediates in exosome (*rrp6*) mutants (5). We found those results to be consistent with our findings in this study. From these data, we conclude that the disruption of Pol I transcription elongation results in defective processing of the nascent rRNA and induces degradation of intermediates.

To evaluate consequences of the *rpa190-F1205H* mutation on the populations of ribosomal subunits, we performed sucrose gradient centrifugation. Sucrose gradient centrifugation provides an overall view of the abundance and activity of cytoplasmic ribosomes involved in translation. This technique is used to separate ribosomal subunits, fully intact ribosomes, and polyribosomes, and the resultant data can be used to make qualitative predictions about the efficiency of ribosome assembly and function in cells. Using this technique, we observed a substantial reduction in the relative abundance of 60S ribosomal subunits (*versus* either 40S subunits or 80S monosomes) in the *rpa190-F1205H* cells compared with WT (Figs. 5*C* and S7). Consistent with the imbalanced proportion of the large subunit population relative to that of the small subunit, we also observed half-mers, which indicate that a small subunit has engaged the mRNA template but has yet to associate with a large subunit. These half-mers appear as shoulder peaks detectable on the monosome and disome peaks (labeled in Fig. 5*C*, model of half-mer formation in Fig. 5*D*). Thus, the appearance of half-mers is consistent with the observation of reduced 60S subunit abundance relative to 40S subunits in the mutant strain.

Sucrose gradient analyses were performed in triplicate, and representative traces are shown in Figure 5*C*. Quantification of sucrose gradient traces is complicated because gradients are loaded for equal amounts of material that absorb UV light at 260 nm. Since many molecules absorb UV, the baseline absorption does not uniquely reflect ribosome abundance. Thus, the absolute amplitude of any position in the trace does not necessarily reflect a biologically significant difference (*e.g.*, the apparent increase in polysomes in the mutant trace). When the baseline is calculated by extrapolating between “valleys” in the trace, we determined that there was approximately a fourfold decrease in the average 60S abundance relative to 40S in the mutant strain, as compared with an almost threefold increase in WT yeast (Fig. S7). These observations demonstrate that in *rpa190-F1205H* yeast, the production of the 60S subunit is preferentially impaired compared with the 40S, which leads to inefficient translation initiation. Altogether, these findings demonstrate that transcription elongation is directly coupled to rRNA processing and ribosome assembly.

## Discussion

### Pol I transcription elongation properties directly affect ribosome biogenesis

Cotranscriptional processing of nascent RNA has been appreciated for many years (*e.g.*, the 5′ capping of mRNA). Interestingly, the elongation phase of transcription has also been implicated in events such as alternative splicing (8, 28, 29). Pre-rRNA processing serves as an excellent model system to study the connection between transcription elongation properties and RNA processing efficiency because of its cellular abundance, intimate connection to cell viability, and clearly defined cotranscriptional nature. In this study, we used a Pol I TL mutant, *rpa190-F1205H*, which exhibits a reduction in transcription elongation rate (17). Characterization of the transcription elongation properties of *rpa190-F1205H* Pol I revealed an increased tendency to pause both *in vitro* and *in vivo*. Consistent with previous studies investigating elongation-defective Pol I mutants (5), rRNA processing and ribosome biogenesis were impaired by this mutation. These results suggest that the transcription elongation properties of Pol I have coevolved with early rRNA processing steps to promote efficient cotranscriptional processing of rRNA and ribosome assembly.

Previous results suggested that Pol I transcription elongation directly influences the efficiency of ribosome biogenesis (30, 31, 32). A critical finding in support of this idea was the characterization of an elongation-impaired Pol I mutant, *rpa135-D784G* (5). This mutant exhibited a ∼10-fold defect in transcription elongation rate *in vitro* compared with WT Pol I. Furthermore, in the *rpa135-D784G* yeast strain, there was aberrant rRNA processing and defects in ribosome biogenesis (evidenced by half-mers and a reduced 60S/40S ratio). A separate study demonstrated that deletion of the gene that encodes for the Rpa49 subunit of Pol I also resulted in accumulation of half-mers and a reduction in 60S compared with 40S subunit abundance (20). Those findings showed that *rpa49Δ* Pol I had a reduced elongation rate as compared with WT, which resulted in increased spacing between polymerases and suggested additional torsional stress in the rDNA. In WT yeast cells, Pol I occupancy on the rDNA is high and the polymerases are densely packed, with about 50 elongation complexes per 35S gene (33). The close proximity between Pol I elongation complexes prevents accumulation of positive supercoils in front and negative supercoils behind each polymerase. Positive supercoiling in front of polymerases could present an obstacle during transcription elongation, and severe supercoiling could even inhibit transcription altogether. These data highlight an important question: is the elongation rate, pausing propensity of Pol I, or spacing between polymerases important for efficient rRNA processing and ribosome biogenesis? One study was performed to test this *via* mutation of an important preinitiation complex factor, Rrn3. The findings from that study demonstrated that increased spacing between polymerases (because of impaired transcription initiation) does not lead to ribosome assembly defects (5). Therefore, we propose that transcription elongation properties rather than Pol I spacing are critical for cotranscriptional processes. Along these lines, the effect of a Pol I mutant that exhibits a gain-of-function phenotype (such as increased elongation rate) on ribosome biogenesis would be a further test of this hypothesis. Indeed, one would hypothesize that an alteration of elongation rate through either a decrease (as shown here) or an increase would negatively impact rRNA processing and ribosome biogenesis. Interestingly, a hyperactive Pol I mutant has been described in the recent literature (34). Those findings demonstrated that the Pol I mutant, *rpa135-F301S*, produced more rRNA overall compared with WT Pol I both *in vitro* and *in vivo*, but the effects of this mutation on rRNA processing have not yet been characterized.

### Which features of transcription elongation influence rRNA processing?

The goal of this study was to investigate the importance of elongation rate *versus* pausing with respect to cotranscriptional events; however, the two features are difficult to disentangle. To influence transcription elongation rate directly, the Pol I catalytic functions must be impaired. As a result, we expect most elongation-defective enzymes to exhibit altered pausing properties as well. In addition, any increase in pausing will inherently decrease overall transcription elongation rate. Interestingly, the *rpa190-F1205H* mutation did not simply increase the dwell time of pause sites already exhibited by the WT enzyme *in vivo*, rather, the mutation resulted in a redistribution of pausing sites with preference for the 5′ end of the rDNA template (Fig. 3). Based on these data, we conclude that the introduction of abnormal pause sites for Pol I, together with a modest defect in transcription elongation rate, causes severe defects in rRNA processing.

NET-Seq has been used to identify sequence features that govern pausing by Pol II (35) and by *Escherichia coli* RNA polymerase (36). However, such sequence motifs have not yet been definitively identified for Pol I, possibly because Pol I transcribes a single ∼6 kb gene as compared with Pol II, which transcribes thousands of genes. Using NET-Seq in this study, we have determined that there is repositioning of Pol I in the top 2.5% most occupied positions for the WT *versus rpa190-F1205H* yeast (Fig. 4). Our findings demonstrate that in the spacer regions, *rpa190-F1205H* Pol I preferentially occupies A/G-rich regions, as opposed to the C/T-rich regions occupied by WT Pol I. Indeed, both EM of Miller chromatin spreads and NET-Seq revealed dramatic redistribution of Pol I occupancy as a consequence of a single point mutation in the TL region. The findings from this study as well as previous publications (4, 5) demonstrate that there is a clear link between transcription elongation and rRNA processing, and it is reasonable to hypothesize that this relationship could at least partially rely on rDNA template sequence content. However, the role of sequence-specific effects in regulating this relationship is still not well understood. A recent publication suggested that template sequence could affect the pausing propensity of Pol I and possibly folding of the rRNA (9); however, the coordination of these processes remains largely undefined.

### Does Pol I pausing facilitate cotranscriptional ribosome assembly?

This study focused on the characteristics of the *rpa190-F1205H* Pol I and how defective transcription elongation properties can perturb rRNA processing events. Since NET-Seq data can be viewed as a snapshot of Pol I occupancy *in vivo*, one may interpret large peaks as sites of polymerase pausing. Therefore, Figure 3 demonstrates the heterogeneity of Pol I occupancy *in vivo*, consistent with previous findings (9, 22, 23, 24, 25, 37), and indicates that Pol I faces barriers to transcription elongation, even though the rDNA is generally thought to be mostly free of phased nucleosomes (38). Whereas these data highlight an apparent nucleotide incorporation preference and multiple sites of increased Pol I occupancy throughout the 35S gene, there are many open questions: do these pause sites play a role in cotranscriptional or post-transcriptional processing? If so, are they conserved among eukaryotic species? We posited previously that because sequence similarity and secondary structure proximal to the active site are highly conserved throughout both eukaryotic and prokaryotic DNA-dependent RNA polymerases, sequence-dependent pausing effects observed in yeast Pol I might be conserved in other polymerases as well. On the other hand, the primary sequence for the rDNA in various species can differ a great deal (though core mature secondary structure is largely conserved). Are sequence effects consistent for Pol I throughout Eukarya? All these questions are raised by the novel capabilities provided by experimental approaches like NET-Seq. As these questions are answered over time, we expect to determine the significant evolutionary constraints applied to the rDNA to optimize ribosome function as well as assembly and potentially regulation of ribosome biosynthesis.

### Are trans-acting factors involved in coordinating Pol I elongation and cotranscriptional ribosome assembly?

These data suggest that Pol I elongation kinetics are important for cotranscriptional processes; therefore, it is conceivable that the actions of transcription factors, which modulate Pol I elongation, may also affect ribosome assembly. Indeed, one such factor might be Paf1. It was previously established that the deletion of genes that encode Paf1 subunits leads to reduced Pol I elongation rate and aberrant cotranscriptional rRNA processing (19). In addition, Spt4 and Spt5 have been shown to influence Pol I transcription elongation *in vivo* (23), and deletion of *SPT4* results in a reduction in Pol I elongation rate and rRNA processing defects (39). These studies suggest that various transcription factors may facilitate proper elongation and possibly cotranscriptional ribosome assembly.

The effects of transcription factors on pausing have previously been shown to alter the folding of RNA cotranscriptionally. In *E. coli*, the folding of RNAse P domains was influenced by the transcription elongation factor NusA (10). These data suggest that transcription factors can directly affect cotranscriptional RNA processing and maturation. It is reasonable to suggest that in cells, transacting factors might manipulate pause probability or rates of nucleotide addition to tune the assembly or activity of processing factors on the nascent rRNA. Currently, we have only begun to reveal the complex relationship between transcription elongation and the myriad of steps required to build functional eukaryotic ribosomes.

## Experimental procedures

### Yeast strains, plasmids, and growth conditions

Yeast cultures were grown at 30 °C in standard growth conditions, and yeast strain manipulation was performed using standard methods (40). For isolation of *rpa190-F1205H*, the mutation was introduced into a pBlueScript derivative carrying a region of *RPA190* using PCR-directed mutagenesis. Sanger sequencing was used to validate the construct. The validated mutant plasmid was cloned into pRS315 containing the entire *RPA190* gene flanked with 500 base pairs both upstream and downstream. The mutant plasmid was then transformed into a diploid strain, *RPA190*/*rpa190Δ*::HIS3, and sporulated. Haploids were isolated by dissection and selected for on SD -Leu -His double dropout media to identify *rpa190Δ*::*HIS3* <pRS315-*rpa190-F1205H*> strains. For the *RPA135-FLAG* and *RPA135-HA* strains, the tag was introduced into the genomic *RPA135* locus by recombination (40).

### *In vitro* transcription

Pol I was purified, and *in vitro* experiments were performed as previously described (17, 18). Briefly, the template sequence included the native promoter and rDNA sequence, except for a stretch of six Gs in the template strand. This enables synchronization of the polymerases at that position with the omission of CTP and is key for elongation rate assays. The sequences used *in vitro* were cloned from positions in the rDNA 5′ ETS (+405 to +604) and 18S (+2246 to +2445) and placed immediately downstream of the native rDNA promoter and the initially transcribed 55-nucleotide C-less stretch. The rDNA position is numbered with respect to the transcription start site. *In vitro* reactions (20 μl each) were performed in 1× transcription buffer (20 mM Tris–OAc [pH 7.9], 100 mM potassium glutamate, 8 mM magnesium acetate, 2 mM DTT, RNasin Plus [Promega], 0.2 mg/ml acetylated bovine serum albumin), 10 μCi of [α^32-^P] UTP (PerkinElmer), 10 μM ultrapure NTPs (GE Life Sciences), and quenched with 1 ml of 1.25 M ammonium acetate in 95% ethanol.

### Miller chromatin spreads

EM of Miller chromatin spreads was performed as previously described (33), and quantification of gaps was performed as described (19). Briefly, a “large gap” was defined as a stretch of rDNA that could accommodate 40 polymerases (∼25% of the total rDNA length). The position of the start (promoter-proximal edge) of the gap was determined. The data were binned in intervals of 10% of the gene length (∼670 bp/bin), and the frequency of observing the start of a gap within each bin was plotted at the position of the midpoint of the bin. The rDNA repeats shown are representative of the populations observed from mutant or WT cells.

### NET-Seq

NET-Seq was performed exactly as previously described (25) using the *rpa190-F1205H* strain with an additional 3×-hemagglutinin tag on the C terminus of Rpa135 and the appropriate parent strain. Briefly, 1 l cultures (a total of 3 l per strain) were harvested and lysed under cryogenic conditions. Pol I elongation complexes were immunoprecipitated, and the RNA was extracted. A DNA linker containing a unique molecular identifier was ligated onto the 3′ end of the isolated nascent transcripts (23) to preserve the last incorporated nucleotide position and then reverse transcribed. Finally, the complementary DNA was circularized and amplified *via* PCR to produce high-throughput sequencing libraries using a NextSeq 500 as previously described (22, 23). Amplification primers for each sample are included in Table S1.

NET-Seq data analysis was performed exactly as published, using the same options and function settings as previously described (23, 24, 25). In summary, reads were deduplicated based on the unique molecular identifier sequence as previously described using fqtrim (version 0.9.7 (41)). Next, the 5′ and 3′ adaptors were trimmed off of resultant reads using cutadapt (version 3.4 (42)). Reads were aligned to the yeast genome (*S. cerevisiae* genome assembly 64-1-1) using the STAR aligner (version 2.7.1a (43)). Finally, resultant BAM files were sorted and indexed using SAMTools (version 1.6 (44)), converted to BED files, and genome coverage files were generated using BEDTools (version 2.28.0 (45)).

Following genome coverage file generation, all data analyses and visualizations were performed using R (version 4.1.3) and RStudio (version 2022.02.1). An organized data frame containing coordinate number, region identity, nucleotide, and normalized counts was created exactly as previously described (23). Histograms were generated either by using the built-in plotting function of R or with ggplot2 (version 3.3.5), and Spearman correlation coefficients were calculated using R. The DiffLogo package (version 2.18.0) was used to create the DiffLogo plot. A complete list of software packages and versions used is available in Table S2. Raw data can be accessed through the Gene Expression Omnibus of the National Center for Biotechnology Information *via* series accession number GSE196146. The data processing code and R scripts are available upon request.

### Northern blot analysis

RNA was isolated from cells harvested during exponential phase by phenol–chloroform extraction followed by an ethanol precipitation. The RNA pellets were suspended in water, and the concentration of RNA was normalized between samples. Equal amounts of RNA were run on 0.9% formaldehyde:agarose gels and then transferred to a nylon membrane. The Northern procedure was performed using an HB-500 Minidizer hybridization oven (UVP). The membrane was prehybridized at 65 °C for 4 h in 20 ml hybridization buffer (0.5 M sodium phosphate [pH 7.2], 7% SDS [w/v]). Oligo probes (0.5 μM) were labeled using [γ^32^-P] ATP and polynucleotide kinase (NEB) for 1 to 2 h. The entire oligo labeling reaction (20 μl) was added to the hybridization buffer and allowed to hybridize at 65 °C overnight. Prior to washing, the blot was incubated at room temperature for 2 h and then washed in 20 ml wash buffer (40 mM sodium phosphate [pH 7.2] and 1% SDS [w/v]) for 15 min at room temperature, 10 min at 42 °C, and then 15 min at room temperature. The labeled blots were exposed to a phosphor screen and developed using a phosphorimager (Typhoon Trio; GE). Bands were quantified using ImageQuant (GE Life Sciences). Including the sequence of the probes used, this technique was performed as previously described (17). Hybridization probe sequences are included in Table S3, and a probe map can be found in Fig. S6.

### Sucrose gradient sedimentation

The separation of ribosomal subunits and polysomes was performed using sucrose gradient sedimentation. Immediately prior to harvesting, cells were treated with cycloheximide (100 μg/ml, final concentration). Cells were lysed in breakage buffer (20 mM Tris–HCl [pH 7.5], 100 mM NaCl, 30 mM MgCl_2_·6H_2_O, 0.1 mg/ml cycloheximide, and 0.2 mg/ml heparin) using a FastPrep with four cycles at 4.5 m/s. Sucrose gradients were poured in a solution base of 50 mM Tris–HCl (pH 7.5), 50 mM ammonium chloride, 12 mM MgCl_2_·6H_2_O, and 1 mM DTT, with 50%, 45%, 40%, 35%, 30%, 25%, 20%, 15%, 10%, and 5% sucrose (from bottom to top, respectively). Lysates were normalized to RNA concentration (absorbance at 260 nm), loaded onto the top of the sucrose gradient, and spun at 30,000 rpm for 5 h at 4 °C in a Beckman SW41 rotor. Finally, 60% sucrose (pumped through a pierced bottom of the column) was used to push the gradient through an FPLC, and absorbance of the elution was recorded at 254 nm.

## Data availability

All data described within the article are included in this article and supporting information. NET-Seq raw data have been deposited into the Gene Expression Omnibus of the National Center for Biotechnology Information and can be accessed *via* series accession number GSE196146.

## Supporting information

This article contains supporting information (5, 17).

## Conflict of interest

The authors declare that they have no conflicts of interest with the contents of this article.
