# Supplementary material for: Rate of transcription elongation and sequence-specific pausing by RNA polymerase I directly influence rRNA processing
Source: J Biol Chem. 2022 Nov 22;298(12):102730. doi: 10.1016/j.jbc.2022.102730 (PMC9768379; doi:10.1016/j.jbc.2022.102730)
Supplement: Supplemental Figures S1–S7 and Tables S1–S3 [file mmc1.docx]

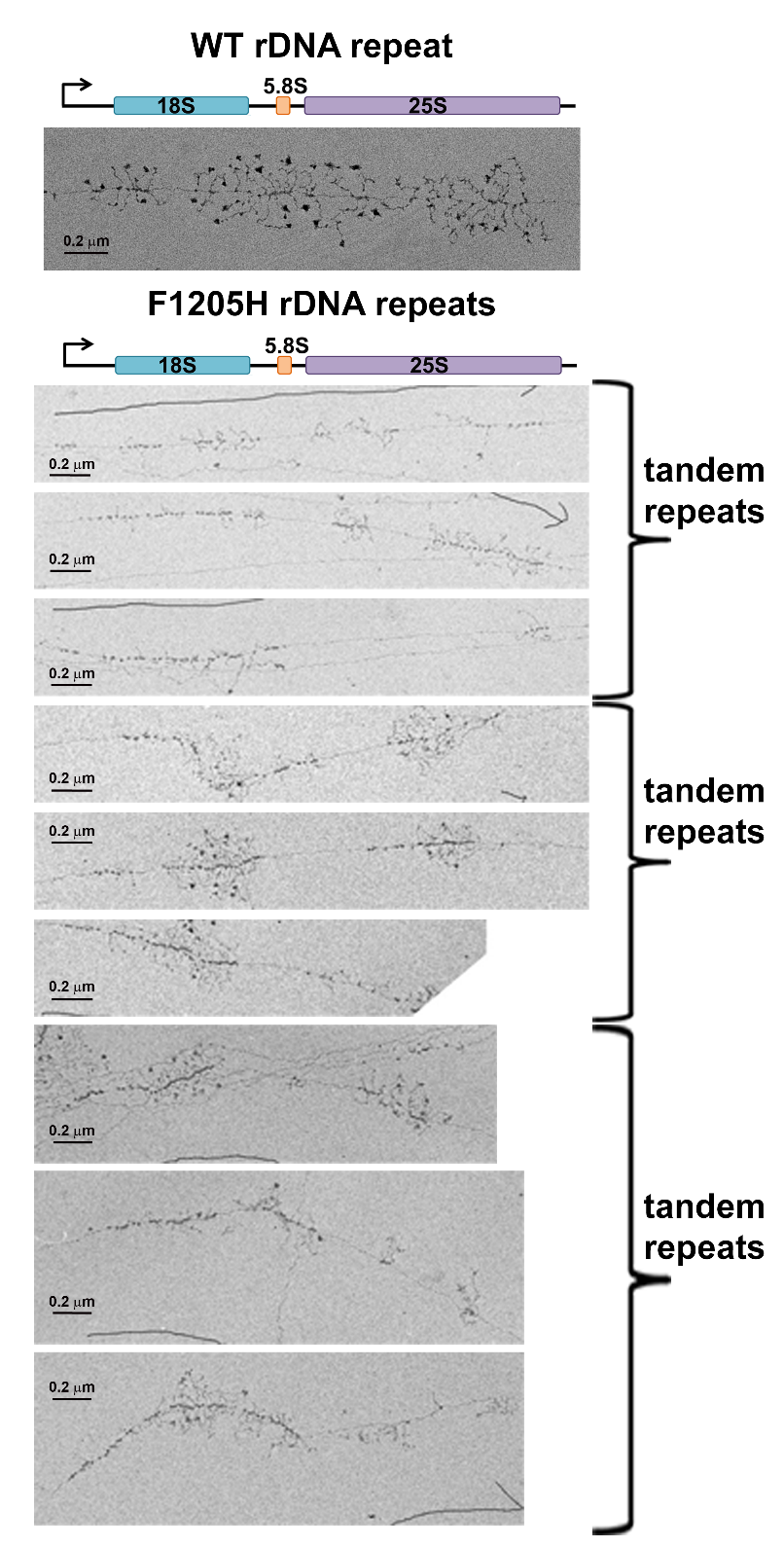


**Supplemental Figure 1. Additional Miller chromatin spreads for *rpa190-F1205H* yeast.** Several additional rDNA repeats observed in *rpa190-F1205H* yeast are displayed below a single WT repeat for comparison. The same WT repeat is included in figure 2 of the main text. Large gaps are present between transcribing polymerases in the mutant. Bracketed images are tandem repeats in which the rDNA template can be directly traced between the repeats shown. Defects in polymerase occupancy and position are evident in all of the repeats shown. Two of these rDNA repeats from the *rpa190-F1205H* strain are included in the main text figure 2.


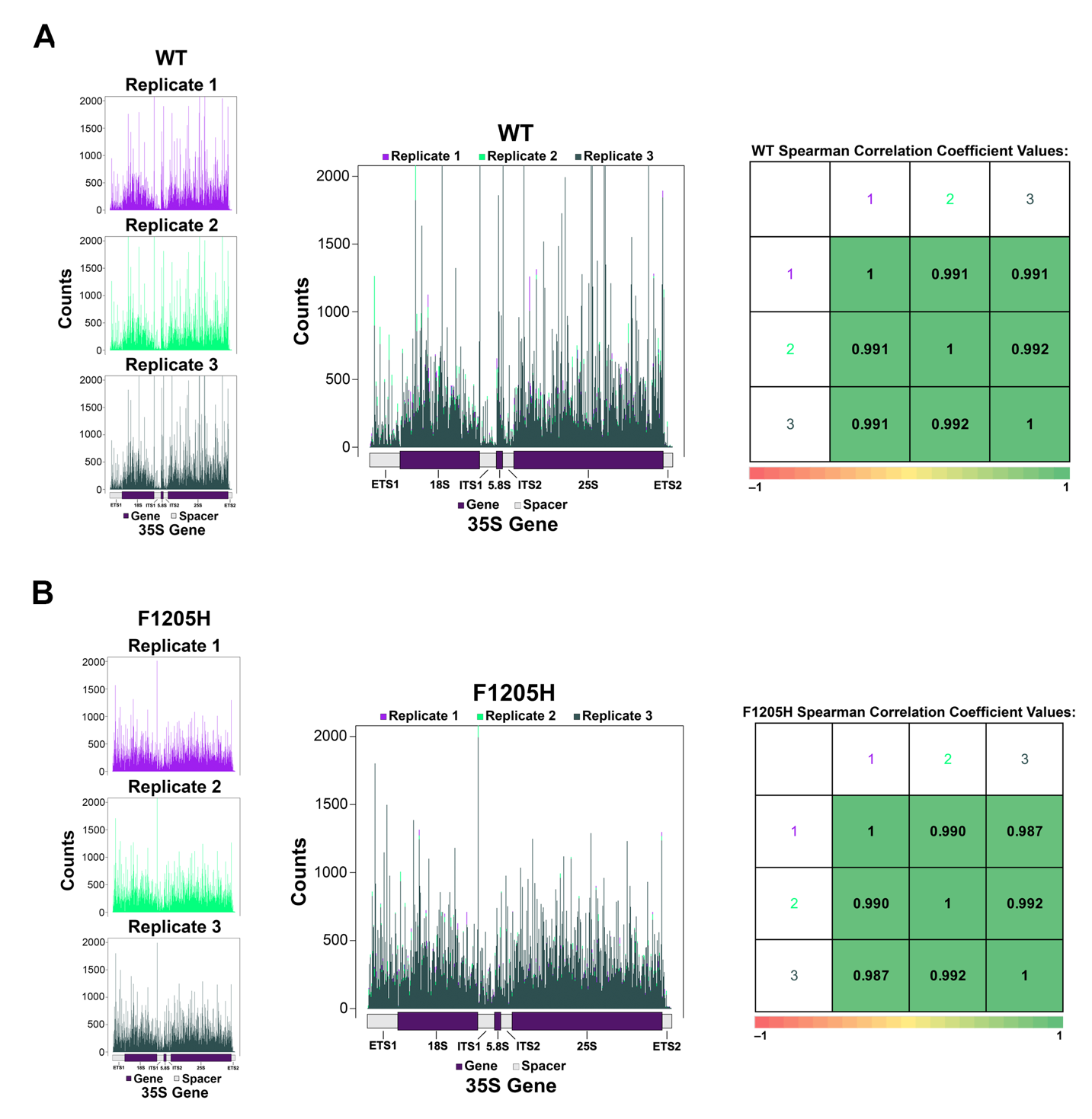


**Supplemental Figure 2. NET-seq experiments for WT and *rpa190-F1205H* yeast strains were highly reproducible.**

NET-seq was performed in triplicate for both WT and *rpa190-F1205H* yeast strains. The 3’ ends of nascent rRNA transcripts were mapped to the yeast genome and plotted for all three replicates (**A and B**, left panels), with replicates overlaid in middle panels. The Spearman correlation test was used to determine the similarity between the three replicates of each strain, where a coefficient value = 1 indicates 100% similarity (**A and B**, right panels)). WT data are displayed in the top panels and *rpa190-F1205H* data are displayed in the bottom panels.


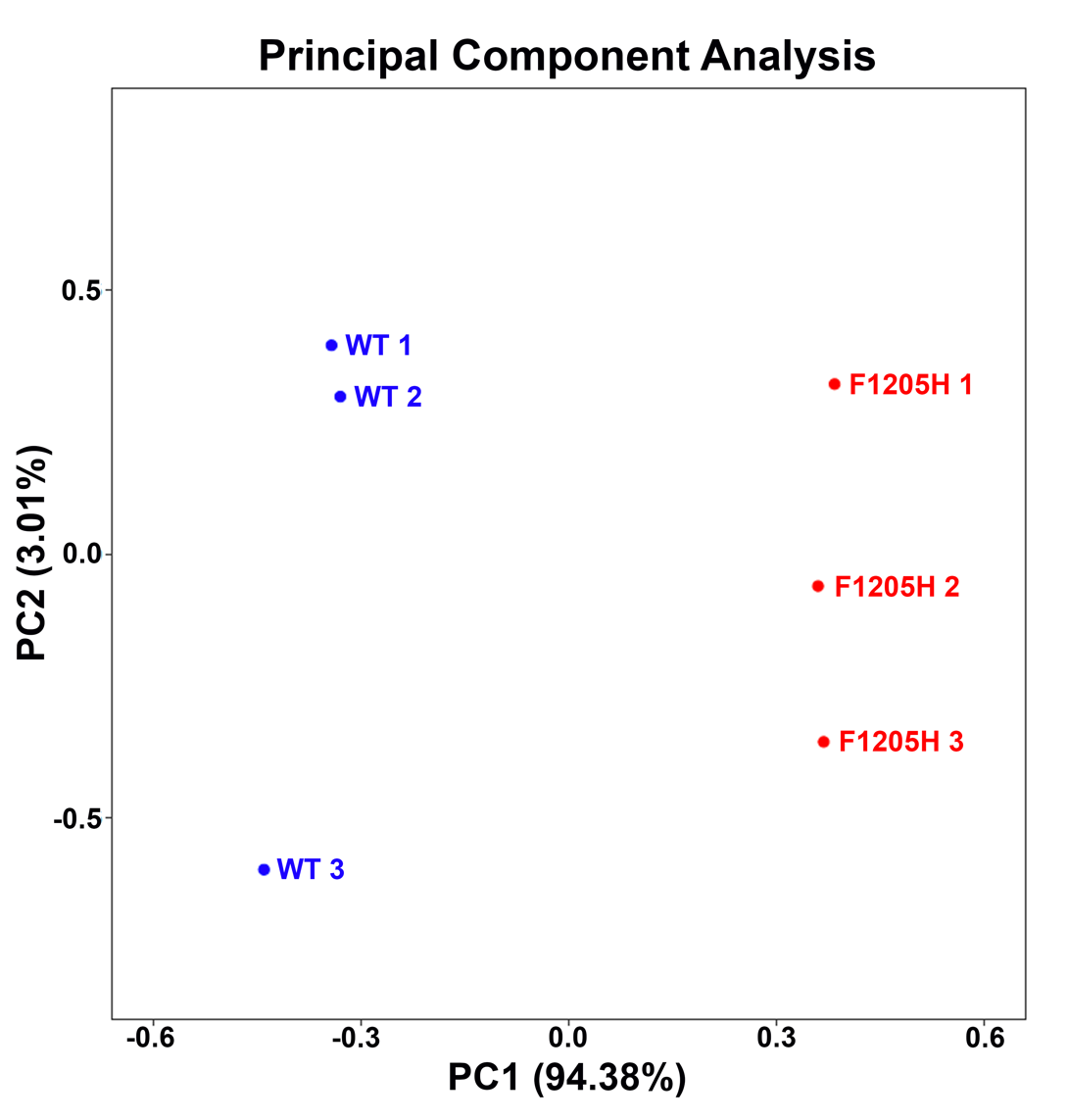


**Supplemental Figure 3. Principal component analysis (PCA) plot shows clustering of samples and indicates dissimilarity between strains.**

PCA was performed, and the results were plotted. The WT samples are displayed in blue, while the *rpa190-F1205H* samples are displayed in red.


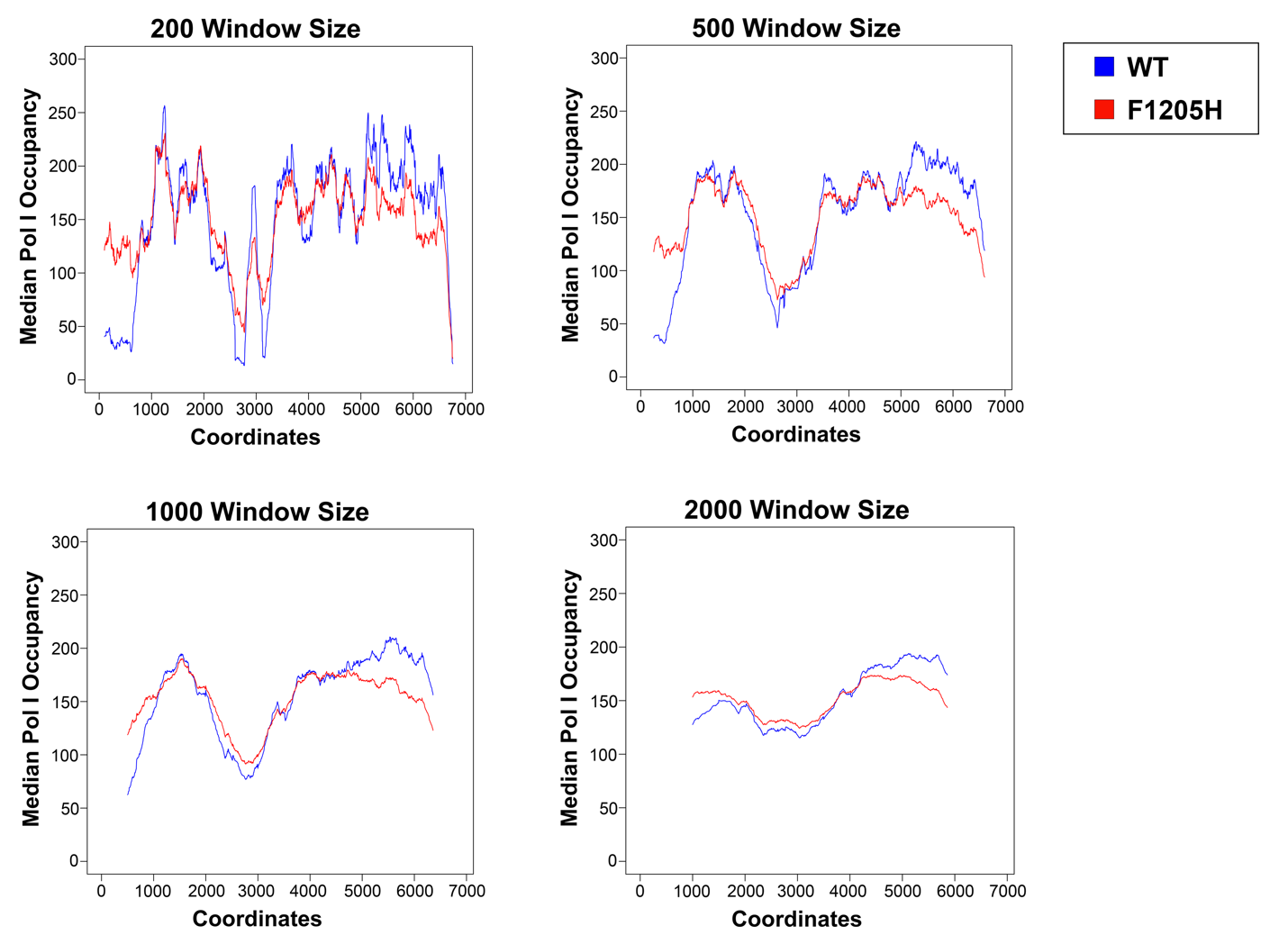


**Supplemental Figure 4. Moving average plots confirm that Pol I occupancy is altered in *rpa190-F1205H* yeast.**

Moving average plots were generated for different window sizes to compare occupancy patterns between the WT and *rpa190-F1205H* strains. The window size indicates the number of positions that the moving average was calculated across. For example, in the 200 window size plot, the first data point is the moving average calculated across the first 200 positions, and so on.


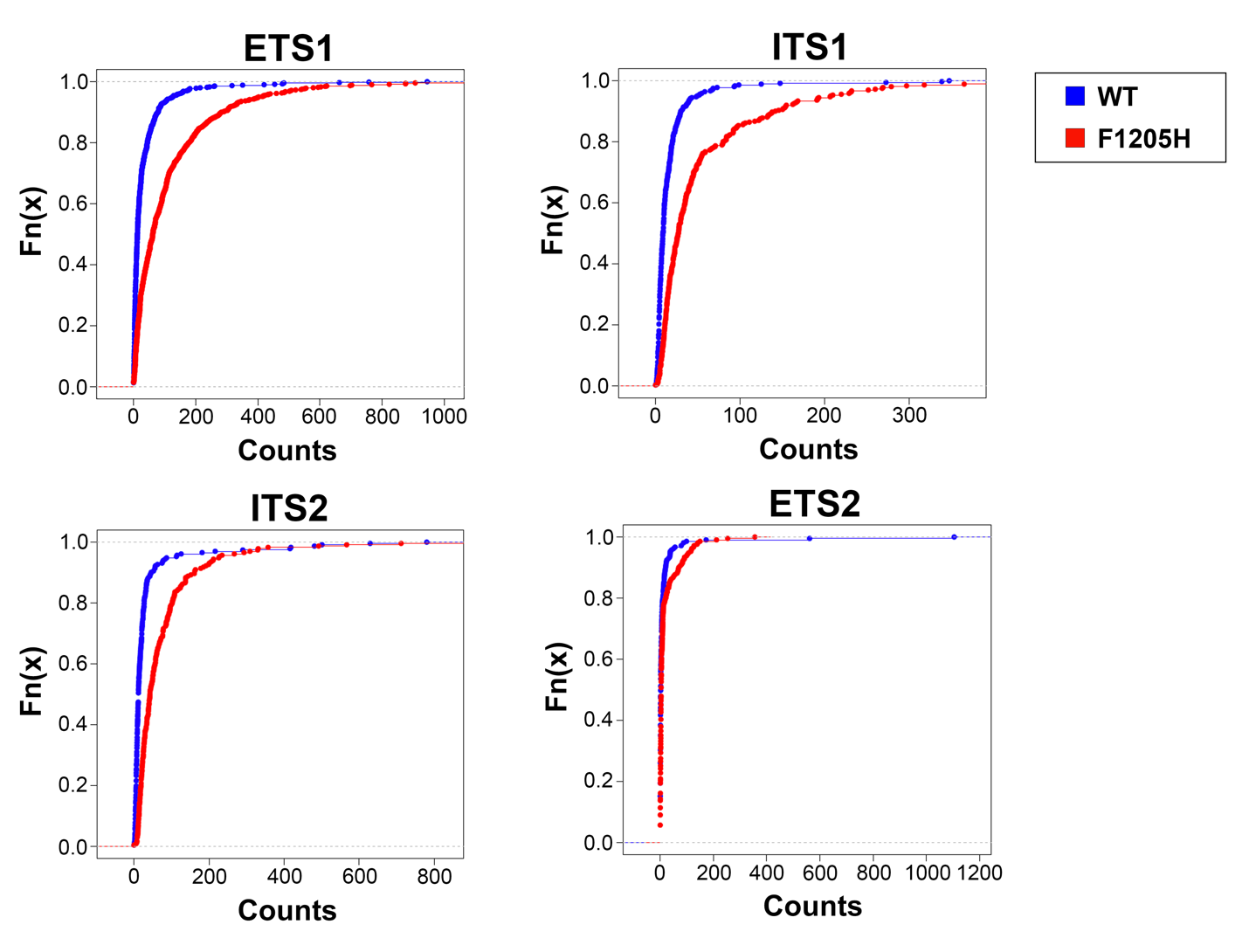


**Supplemental Figure 5. Cumulative distribution function (CDF) plots demonstrate that occupancy differences between WT and *rpa190-F1205H* yeast are most pronounced at the 5’ end of the rDNA template.**

CDF plots were generated to further analyze differences between the WT and *rpa190-F1205H* strains in each of the four spacer regions. The greatest difference between strains can be seen in the most 5’ region (the ETS1), and this difference drops off as the polymerases approach the 3’ end of the rDNA template (see the ETS2).


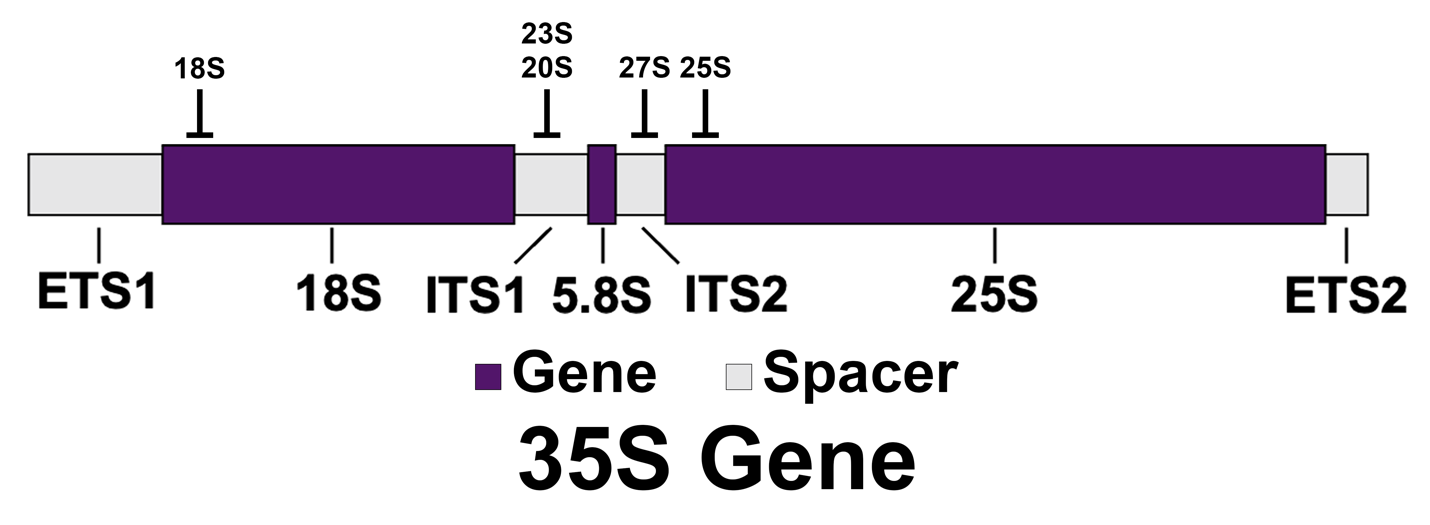


**Supplemental Figure 6. Map of Northern blot probe targets on the rDNA.**

Target locations (18S, 20S, 23S, 27S, and 25S) for the Northern blots displayed in Figures 5A and B are mapped to the 35S gene.


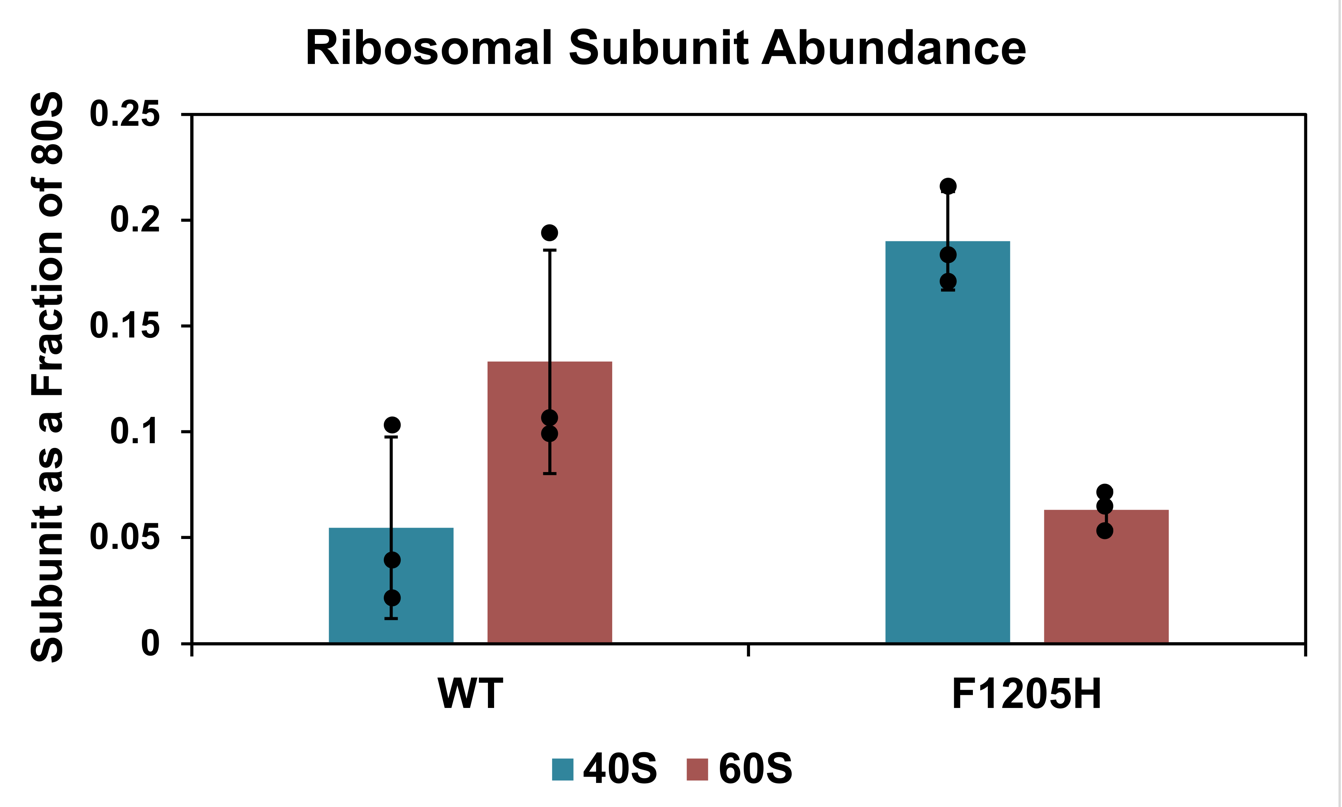


**Supplemental Figure 7. Quantification of sucrose gradient analyses indicate a decrease in the relative abundance of 60S to 40S in the mutant vs. WT.**

The abundance of 40S and 60S subunits as a fraction of 80S were calculated across three biological replicates. Error bars demonstrate the standard deviation between data points and raw data points are included for each subunit analyzed.

| **Sample** | **Forward** | **Reverse** |
| --- | --- | --- |
| WT1 | CAAGCAGAAGACGGCATACGAGATgctcaggaTCCGACGATCATTGATGGTGCC | AATGATACGGCGACCACCGAGATCTACACtagatcgcCGTCTCTTCTGCGGATGACTCG |
| WT2 | CAAGCAGAAGACGGCATACGAGATaggagtccTCCGACGATCATTGATGGTGCC | AATGATACGGCGACCACCGAGATCTACACtagatcgcCGTCTCTTCTGCGGATGACTCG |
| WT3 | CAAGCAGAAGACGGCATACGAGATcatgcctaTCCGACGATCATTGATGGTGCC | AATGATACGGCGACCACCGAGATCTACACtagatcgcCGTCTCTTCTGCGGATGACTCG |
| MUT1 | CAAGCAGAAGACGGCATACGAGATgtagagagTCCGACGATCATTGATGGTGCC | AATGATACGGCGACCACCGAGATCTACACtagatcgcCGTCTCTTCTGCGGATGACTCG |
| MUT2 | CAAGCAGAAGACGGCATACGAGATcctctctgTCCGACGATCATTGATGGTGCC | AATGATACGGCGACCACCGAGATCTACACtagatcgcCGTCTCTTCTGCGGATGACTCG |
| MUT3 | CAAGCAGAAGACGGCATACGAGATagcgtagcTCCGACGATCATTGATGGTGCC | AATGATACGGCGACCACCGAGATCTACACtagatcgcCGTCTCTTCTGCGGATGACTCG |

**Supplemental Table 1. NET-seq library amplification primers.**

Primer sequences are included for all WT (WT1-3) and *rpa190-F1205H* replicates (MUT1-3).

| **Software** | **Version** |
| --- | --- |
| fqtrim | 0.9.7 |
| cutadapt | 3.4 |
| FastQC | 0.11.7 |
| Anaconda | 5.3.1 |
| STAR | 2.7.1a |
| SAMTools | 1.6 |
| BEDTools | 2.28.0 |
| R | 4.1.3 |
| RStudio | 2022.02.1 |
| dplyr | 1.0.8 |
| plyr | 1.8.7 |
| ggplot2 | 3.3.5 |
| ggseqlogo | 0.1 |
| ggpubr | 0.4.0 |
| cowplot | 1.1.1 |
| matrixStats | 0.62.0 |
| hexbin | 1.28.2 |
| tweedie | 2.3.3 |
| statmod | 1.4.36 |
| magritter | 2.0.3 |
| scales | 1.2.0 |
| tidyr | 1.2.0 |
| zoo | 1.8-10 |
| DiffLogo | 2.18.0 |
| rclone | 1.48.0 |

**Supplemental Table 2. Software packages and versions used in NET-seq data analysis.**

| **Location** | **Probe Sequence** |
| --- | --- |
| 20S and 23S | 5’- GCACAGAAATCTCTCACCGT -3’ |
| 27S | 5’- GCCTAGACGCTCTCTTCTTA -3’ |
| 18S | 5’- AGCCATTCGCAGTTTCACTG -3’ |
| 25S | 5’ -ACTAAGGCAATCCCGGTTGG -3’ |

**Supplemental Table 3. Sequences of Northern blot probes.**

Hybridization probes used in this study were first described in (1) for the 20S, 23S, and 27S and (2) for the 18S and 25S probe locations.

**REFERENCES**

1. Schneider, D. A., Michel, A., Sikes, M. L., Vu, L., Dodd, J. A., Salgia, S., Osheim, Y. N., Beyer, A. L., and Nomura, M. (2007) Transcription elongation by RNA polymerase I is linked to efficient rRNA processing and ribosome assembly. *Mol Cell* **26**, 217-229

2. Viktorovskaya, O. V., Engel, K. L., French, S. L., Cui, P., Vandeventer, P. J., Pavlovic, E. M., Beyer, A. L., Kaplan, C. D., and Schneider, D. A. (2013) Divergent contributions of conserved active site residues to transcription by eukaryotic RNA polymerases I and II. *Cell Rep* **4**, 974-984
